# And the award goes to…the Matthew Effect: Examining external status as a predictor of productivity and opportunity

**DOI:** 10.1371/journal.pone.0290954

**Published:** 2023-10-24

**Authors:** Molly E. Contini, Jeffrey R. Spence

**Affiliations:** Department of Psychology, University of Guelph, Guelph, Ontario, Canada; National Taiwan University, TAIWAN

## Abstract

It has been suggested that increased status that comes from being an award winner can generate enduring advantages that compound over one’s career via the Matthew Effect. However, research in this area has yielded conflicting results and has been unable to isolate the unique effect of status on career outcomes from the positive endogenous characteristics of award winners. In the current research, we attempt to address previous research limitations and examine if winning an award is associated with career outcomes (i.e., opportunities and productivity) irrespective of individual productivity levels prior to receiving an award. We examined our research questions using observational data of National Hockey League (NHL) league championship winners and non-winners (*N* = 427). By using a team award and several different analytic approaches we were able to examine the unique effects of affiliation-based external status, generated from an award win, on career outcomes. Our results generally show support for the Matthew Effect and suggest that affiliation-based external status, achieved by an award win, provides access to increased opportunities, which ultimately results in more productivity. We discuss the importance of incorporating opportunity and investigating its role in the cumulative advantage process and implications of the results.

## Introduction

Awards are prevalent in almost every area of life from sports, the arts, culture, politics, academia through to the corporate domain [1]. Awards can be awarded to organizations, teams or individuals and can recognise a single event or an enduring activity [2]. Although awards can differ in several aspects, they fundamentally serve to recognize and distinguish an individual, team, or organization on criteria deemed important by the award giver [3]. Additionally, awards can be used as information to help gatekeepers make decisions in a variety of selection/assessment contexts (e.g., scholarship applications, hiring decisions). However, despite the ubiquity of awards and their regular use in assessment, exactly how awards may affect individuals’ career outcomes is still unresolved.

R.K Merton famously conceptualized “the Matthew Effect” and has led the investigation into the impact of awards on career outcomes. The Matthew Effect posits that increased status garnered from winning an award is a resource in and of itself [4]. Over time, status is thought to lead to successively greater opportunities for advancing work, which, in turn, results in greater productivity and the rewards that go with increased productivity [5]. In line with this perspective, there is evidence to suggest that winning an award positively impacts future productivity and results in more rewards [6,7]. At the same time, there is also evidence to suggest award winners are less productive after the receipt of an award [8,9]. These conflicting findings raise questions about the appropriateness and efficacy of using awards to make decisions about individuals.

The current research aims to clarify the way status, accrued by an award win, influences career outcomes in two main ways. First, this work isolates and investigates the main mechanisms of the Matthew Effect. Recall, the Matthew Effect also proposes that status results in increased opportunities (e.g., job offers, chances for collaborations), which are said to result in more productivity and career success [4]. However, many studies that investigate the Matthew Effect strictly compare productivity metrics pre and post award [9,10] and the extent to which status results in inflated career opportunities is not known. As such, it is challenging to pinpoint where discrepancies in career outcomes between winners and non-winners begin to occur, and how differences in career paths develop and operate.

Secondly, this work acknowledges and attempts to resolve another important complication that is inherent to awards: namely, awards are often conflated with positive endogenous characteristics of award holders. That is, because awards are generally thought to be bestowed to the top candidate in a domain, it is difficult to determine the extent to which career outcomes may be due to the positive individual characteristics that resulted in receiving the award (e.g., talent, ability) or are the result of status and reputation gains that came from receiving the award. Specifically, if award winners are found to have better career outcomes than non-winners, are the outcomes attributed to the award itself or to the positive qualities of the winner that were in place prior to receiving the award?

Accordingly, the goal of the current paper is to test if benefits from receiving an award (e.g., status, reputation) are predictive of positive career outcomes, irrespective of recipients’ exceptional endogenous characteristics (e.g., talent, ability). Together, this work contributes to the understanding of the Matthew Effect and the impact of awards on career outcomes by isolating the unique effect of awards from recipients’ positive endogenous characteristics and investigating the key components of the Matthew Effect, including opportunities, as possible mechanisms.

### Awards and status

In most cases, awards are markers of excellence and are celebrated and announced in a public fashion [3]. Winning an award sets the recipient apart from their peers and can have positive implications for their careers. Often, winning an award results in a special form of social distinction that can spread and grow over time [11]. As long as prospective recipients value the award and have a desire to obtain it, recipients can enjoy status and reputational and recognitional benefits as a result of being connected to the award [1,11]. This recognition may be quite enduring, impactful, and can even grow as more people become aware of and recognize a winner’s distinction.

Recognition that is conferred by the eminence of the award has also been referred to as *external status*. External status can be defined as how broad one’s visibility is outside of their own organization [12]. Employees attain external status as the result of attributes independent of the level or visibility of their current task performance [12]. A sub-type of external status, affiliation-based external status, does not stem from a demonstrated quality or level of performance, but rather refers to status that is inherited as a result of a close personal connection with and/or sponsorship by an elite individual or institution [12,13]. Affiliation-based external status can be considered the reputational benefits and favorable attributions one is ascribed on the basis of their associations (e.g., the prestige of the university they attended, the notoriety of an award they won). The exclusivity and reputation of the affiliation is thought to be correlated with the value gained by the affiliation [12,13]. Namely, affiliations perceived as prestigious will result in more status than lesser-known or easier to obtain affiliations.

The benefits of having affiliation-based external status are numerous. Affiliation-based external status serves as a signal or message to outsiders, regardless if they are a subject matter expert or not, that the award winner is admirable [3]. The heightened perception of the individual, in turn, influences perceptions of the quality of the work the individual produces [14]. Status creates the assumption of higher quality and may implicitly result in greater attention to the work of those with status [4]. Thus, award winners can receive more favourable judgments as a result of their affiliation-based external status and this can result in more positive career outcomes through increased attention and access to resources.

### Opportunity, productivity and the Matthew Effect

Being the recipient of an award and benefitting from its positive effects is consistent with the Matthew Effect [4,5,13]. The Matthew Effect proposes that receiving an award can potentially create inequalities between two individuals of relatively similar ability by way of its conferred status over time. When award-winning scientists may begin to receive peer recognition, the process of cumulative advantage is thought to begin. In a process similar to compound interest, an initial comparative advantage can compound to yield larger, successive increments of advantage such that the gaps between those who have resources and those who do not widen over time [15,16]. Increased recognition may lead to successively greater opportunities for advancing work, which, in turn, results in greater productivity and the rewards that go with increased productivity [5]. Thus, an initial award win may result in winners, when compared to a finalist for that same award, being more productive and earning more resources over the course of their career as a result of a potentially small initial advantage.

Numerous analytical approaches have been utilized to study the Matthew Effect and its core tenets. Often, researchers contrast award winners’ post-award productivity with a control group of non-award-winning researchers of commensurate abilities [8,9,14]. Prior empirical work has applied matching techniques and have matched either work authored by an award winner [17] or award-winning authors themselves [10] with controls and compared post-award productivity. Researchers have also utilized within-person approaches: some have contrasted individual author’s pre and post award career outcomes [18] and others have utilized formal statistical tests (e.g., Gilbrat’s law; the Kolmogorov–Smirnov test) to analyze citation patterns of authors for post-award deviations [19,20]. Other work has investigated how awards impact the attainment of more objective career success measures such as promotions or salary increases [2,7]. Interestingly, previous findings on how receiving an award influences future productivity have been mixed [6–10] and little is known about how components of the Matthew Effect, specifically opportunity, operate.

More specifically, there are findings that both support and contradict the Matthew Effect being initiated by winning an award. Some research supports the idea that winning an award facilitates subsequent productivity (e.g., publications, future awards) [6,7,18], but there is also evidence to suggest award winners are actually less productive post-win [8–10,17]. Also, despite the importance of opportunities in the Matthew Effect, it is not clear if winning an award affects subsequent opportunities and in turn, how opportunities influence productivity and future rewards. Studies evaluating the Matthew Effect frequently compare measures of post award productivity and often do not gather nor evaluate measures of opportunities pre and/or post award [9,10]. Together, previous empirical research suggests that the effect of awards on recipients’ career outcomes may not be straightforward, and some unknowns still exist.

### The current study

The effect of awards on career outcomes is likely a dynamic process that unfolds over a long period of time and involves an interaction between a number of psychological, perceptual, and decision-making processes both within and outside of the recipient. One of the main challenges of interpreting empirical results on the effect of awards on outcomes is the confounding nature of award winners’ endogenous characteristics with the receipt of an award. That is, trying to answer the questions of if, and the extent to which, affiliation-based external status from awards and recognitions uniquely contributes to career outcomes is inherently difficult because it is generally recognized that receiving an award is the result of some type of demonstrated excellence. Indeed, most of the research on awards examines awards that are achievement-based (e.g., Fields Medal, Nobel Prize). As a result, winners are presumed to be more deserving of an award than non-winners, via their better performance, achievements, qualifications, etc. Therefore, any subsequent differences in career outcomes between an award winner and non-winners may simply be considered a reflection of individual differences that helped lead to the receipt of the award.

Disentangling the effect of winners’ endogenous characteristics and affiliation-based external status on career outcomes is a noted challenge within the Matthew Effect literature. For example, Bol and colleagues [6] purport that research on the Matthew Effect has been hampered by the possibility that observed cumulative differences in achievement may in fact be the gradual manifestation of interpersonal differences in talent or productivity. Azoulay and colleagues [14] suggest that an individual’s quality and status are typically tightly intertwined. In doing so, they question if status affects outcomes or if status is simply a by-product of an individual’s positive endogenous qualities.

Pinpointing and examining the onset of affiliation-based external status can provide a unique opportunity to assess the Matthew Effect and its particular impact on career outcomes. Recall, affiliation-based external status occurs when an individual enjoys broad attention as a result of a close affiliation with an elite person or institution and is independent of the level or visibility of the recipient’s task performance [4,12]. Whereas most of the literature on the Matthew Effect has examined the impact of status achieved by winning performance-based awards, affiliation-based external status is a specific type of status that can be widely recognized, but can be achieved without demonstrating a high level of performance or productivity in a particular domain. As a result, being able to identify the onset of affiliation-based external status can provide a scenario where one can compare post-status acquisition outcomes against those who did not receive the status, while not conflating outcomes with other endogenous characteristics.

A championship in a team sport is a team award and therefore is not specifically a marker of individual team members’ task performance; yet, the team award provides affiliation-based external status to all individual winners: all players who win are part of a small, elite, recognizable group who have won the championship. Most professional sports leagues (e.g., the National Basketball Association, the National Hockey league) actively work to prevent all of the top players from ending up on the same team by instituting salary management practices (e.g., wage caps) [21]. Whereas differences in career outcomes after winning an individual award are often conflated with ability prior to receiving the award, the structure of professional sports necessitates winning teams having players of differing abilities. Accordingly, both winning teams and non-winning teams consist of a large number of players with variable skill and productivity levels. This allows for naturally occurring control groups, where one can find players from winning and non-winning teams with roughly equivalent endogenous characteristics. Thus, award winners can be matched with non-award winners of commensurate ability and comparisons can be made post-award win to determine the unique impact of the award on career outcomes without conflating ability. Considering league championships are awards that are not allocated solely based on *individual* productivity, but still transmit a substantial amount of status, they may be utilized as a marker of external status that is irrespective of endogenous characteristics prior to winning the award.

Examining the Matthew Effect in the context of professional sports has other advantages. Sports data provide a number of objective measures to study the Matthew Effect: awards, opportunities, and productivity. These data also facilitate the examination of phenomena over time as individual player statistics are recorded and logged across careers. This allows researchers to use behavioral data from real-world contexts and makes it possible to create relatively large samples [22]. The meticulous tracking and recording of statistics over players’ careers and objective measures of affiliation-based external status, individual productivity, and individual opportunity makes this type of data an ideal fit for determining how affiliation-based external status uniquely affects career outcomes of individuals.

The current research provides a mechanistic investigation of Matthew Effect by examining if winning an award that confers affiliation-based external status is associated with differences in career outcomes between those who receive the award and those who do not. In doing so, we address two important limitations noted from previous work. (a) By examining an award that is not conflated with individual’s positive endogenous characteristics it will be possible to address concerns raised when interpreting previous research. (b) Furthermore, the current research will address limitations of previous work by formally operationalizing and investigating opportunity as a mechanism of the effect of awards on career outcomes. Using professional sport data, we examine if being designated as “a winner” (by way of attaining a league championship) is associated with better career outcomes (i.e., opportunities, productivity) irrespective of other individual differences that are known to predict success. Specifically, this work seeks to answer:

*Do individuals with affiliation-based external status have more opportunities across their careers than individuals of equal ability who do not have the same affiliation-based external status*?
*a) Are those with affiliation-based external status more productive across their careers than individuals of equal ability that do not have the same affiliation-based external status*? *b) Does opportunity partially explain differences in career productivity between those with affiliation-based external status and those without it*?

To answer these questions, several analytic strategies were applied to two different datasets. In one dataset, award winners are compared to different groups of non-winners. First, award winners are compared to a group of non-winners on career opportunity and productivity statistics. Next, career outcomes between award winners and award finalists are compared. Then, a matching technique is used to compare winners with peers of similar tenure and ability at the time of the winner’s win. Subsequently, we examine whether opportunity increases are a mechanism for productivity gains using mediational analyses. Lastly, two supplementary analyses with additional data are conducted to further investigate the role of opportunity in the Matthew Effect.

## Method

### About the data

To investigate these questions, data from the National Hockey League (NHL) was used. The league championship trophy of the NHL is called the Stanley Cup. Individual player statistics from National Hockey League (NHL) players drafted in the years 1996–2002 were gathered from a publicly available, online database [23]. First, a list of players selected in the 1996–2002 NHL Entry Drafts was compiled. Next, individual players were searched on Hockey Reference. Players yearly statistics and career statistics were recorded. Specifically, we recorded player’s: draft year, name, if they have ever been a Stanley Cup winner or finalist, if they’ve ever received an All-Star team award, their career length (in seasons), games played, points, and total ice time (see Table 1 for descriptive statistics). Lastly, players were then compared to exclusion criteria (noted below) and deemed eligible or not for our work.

**Table 1:** Descriptive statistics.

Variable	*M*	*SD*
Career Length	9.14	4.45
Games Played	512.72	346.27
Points	205.53	233.58
Total Ice Time	8339.74	6909.63

We had some exclusion criteria for our dataset. Goaltenders were excluded as they have different performance statistics than forwards and defenseman. All of the data utilized in this work pertains to regular season performance and opportunity statistics only. The NHL is structured such that all of the teams in the league play an 82-game regular season. The top performing 16 teams in the regular season advance to the play-offs. The bottom 16 teams in the league are eliminated from contention. As such, players on teams that make the play-offs have the potential to play substantially more games (ranging from 4 to 28 games, per season) and to accrue more points and ice time than players on teams that do not make the playoffs. Accordingly, this work excludes play-off statistics from consideration. Additionally, in order for a player to be recognized as a Stanley Cup winner, a player on the Stanley Cup winning team must have played at least 41 games in a single season. Therefore, players drafted within the 1996–2002 drafts who did not play in at least 41 games in a single season were excluded from the dataset as they were not eligible to have their name on the Stanley Cup. Of the 1896 players drafted in 1996–2002, 427 players were deemed eligible. 74 of those players won the Stanley Cup (17%).

### Measures

#### Affiliation-based status

Indicated whether a player had previously won the Stanley Cup (= 1) or whether they had not won the Stanley Cup (= 0).

#### Productivity

Productivity is defined as a “composite measure of efficiency and effectiveness” [24]. To win a game, a team must score more goals than their opponent and, as a result, the most commonly used productivity outputs in hockey are goals, assists, and points. A player receives credit for a goal every time they shoot the puck into the opposing team’s net. A player earns an assist if they pass the puck to a player who scores or if they pass the puck to the player who passes the puck to the player who scores. We followed the industry (i.e., NHL) convention, which is to calculate points by adding goals and assists together. We use points (Pts) as a measure of productivity.

#### Opportunities

Opportunity is defined as "a favorable set of circumstances to create value” [25]. In hockey, each team typically has 18 players in the lineup. Only five players are permitted to be on the ice at one time. Players do not play the game in its entirety. Games are 60 minutes long and players can be on the ice for just a few minutes or can play as many as 35 minutes each game. Regardless of the amount of ice time a player receives, it is only possible for a player to be productive when they are on the ice. We utilize regular season games played (GP) and total time on ice in minutes (TOI) as measures of opportunity.

## Results

### Comparing winners against non-winners and finalists

Our research questions are specific to affiliation-based external status’s relation with opportunities and productivity. To examine these relations, we utilize several analyses that progress from simple to more complex in an attempt to address different interpretations and rule out possible confounds. We utilize a draft cohort strategy for our initial analyses. Players from the same draft years are roughly the same age and therefore have the same potential for opportunities and productivity and will also play against similar opponents throughout their careers. The first analysis is simple, in which we compared the career level data of Stanley Cup winners to career level data of players in the same cohorts who did not win the Stanley Cup. We used two different comparison groups. Specifically, winners were compared to non-winners (every player from the selected cohorts that never won a Stanley Cup in their career) and then compared to winners to finalists (players that reached the Stanley Cup finals at one point in their career, but never won). Finalists can be considered a closer comparison group than non-winners as they closely approximate winners (i.e., finalists that came as close as possible to winning).

Winners were compared to non-winners and finalists across two opportunity statistics (total ice time and games played) and a productivity measure (points). All comparisons were done using a series of paired *t*-tests. Cohen’s *d*, an effect size that reveals the standardized difference between two means, and corresponding confidence intervals (CI), parameters that measure the precision of the effect size, were also calculated. A commonly used interpretation is to refer to effect sizes as small (*d* = 0.2), medium (*d* = 0.5), and large (*d* = 0.8) based on benchmarks suggested by Cohen [26]. Results are displayed below (Tables 2 and 3).

**Table 2:** Results of t-test and descriptive statistics comparing winners to non-winners.

c	Winners		Non-Winners						
Outcome	M	SD	M	SD	n	*Sig*	*df*	*d*	95% CI for Effect Size
GP	767.03	306	456.61	328.35	427	< .001*	425	0.98	.73, 1.24
TOI	13514.11	7283.08	7237.24	6310.44	427	< .001*	425	0.97	.71, 1.22
Points	325.61	277.12	179.94	215.16	427	< .001*	425	0.64	.39, .89

* *p* < .05.

**Table 3:** Results of t-test and descriptive statistics comparing winners to finalists.

	Winners		Finalists						
Outcome	M	SD	M	SD	n	*Sig*	*df*	*d*	95% CI for Effect Size
GP	776.03	305.99	615.59	379.49	163	0.004*	161	0.46	.15, .77
TOI	13514.11	7283.08	10267.2	7364.32	163	0.005*	161	0.44	.13, .75
Points	325.61	277.12	277.39	301.38	163	0.29	161	0.16	-.14, .47

* *p* < .05.

Overall, these results indicate that when comparing the career totals of winners to non-winners and finalists, winners consistently received more opportunities (i.e., played more games, had more time on ice), but were not always more productive. For opportunity measures, all analyses comparing winners with non-winners and winners with finalists reveal statistically significant and medium to large effect sizes. Winners were found to be more productive (medium to large effect, *d >* .5) in their careers compared to non-winners, but did not have more productive careers than finalists.

### Do teams with the best players win?

To help address the perennial issues of causality in understanding the relation between awards and outcomes, additional analyses were conducted with a modified dataset, where the top performing players were removed. Every year, the Professional Hockey Writer’s Association votes for the best 2 performers over the season at each position. Thus, the 10 best players receive a performance-based award at the end of the season and are classified as All-Stars. In this analysis, anyone who had won an All-Star award (e.g., were one of the 10 best players in the league at any point during their career) was removed from the dataset. A total of 15 players were an All-Star at some point in their career and were subsequently removed from the dataset (N = 412). As a result, these analyses examined if affiliation-based external status can generate differences in career outcomes of middle to low performers. Because the current dataset excludes players that have been recognized as top performers (i.e., award winners due to their productivity) and only includes players who would not normally receive any awards for their performance, it offers a unique opportunity to see if affiliation-based status bequeaths benefits irrespective of the endogenous characteristics of award winners. As in the above analyses, opportunity and performance measures between Stanley Cup winners and non-winners and between winners and finalists were compared using a series of paired *t*-tests. Cohen’s *d* and corresponding confidence intervals were also calculated. Results of the analyses are displayed below (Tables 4 and 5).

**Table 4:** Results of t-test and descriptive statistics comparing winners to non-winners, excluding NHL all-stars.

	Winners		Non-Winners						
Outcome	M	SD	M	SD	n	*Sig*	*df*	*d*	95% CI for Effect Size
GP	737.12	293.45	445.39	316.19	412	< .001*	410	0.93	.66, 1.20
TOI	12334.91	6513.59	6997.11	6040.68	412	< .001*	410	0.87	.60, 1.14
Points	280.42	245.41	166.84	186.75	412	< .001*	410	0.58	.31, .84

* *p* < .05.

**Table 5:** Results of t-test and descriptive statistics comparing winners to finalists, excluding NHL all-stars.

	Winners		Finalists						
Outcome	M	SD	M	SD	n	*Sig*	*df*	*d*	95% CI for Effect Size
GP	737.12	293.45	580.9	354.35	150	0.003*	148	0.48	.15, 80
TOI	12334.91	6513.59	9541.81	6838.52	150	0.01*	148	0.42	.09, .74
Points	280.42	245.41	231.12	232.76	150	0.21	148	0.21	-.12, .53

* *p* < .05.

Together, these results mirror the results of the analyses that were conducted with the top performing players included. Winners received significantly more opportunities than non-winners and finalists and were significantly more productive than non-winners, but not more productive than finalists. Overall, our findings remain consistent regardless of the whether the top performers were included in the comparison or not.

### Do differences in outcomes arise after the win?

By finding opportunity and some productivity differences across careers between those with an award and those without, the above results, appear to suggest some support for Merton’s Matthew Effect. However, a comparison of total career outcomes leaves the results vulnerable to the possibility that differences in productivity and opportunity are not the result of increased affiliation-based external status. That is, the differences in career totals could have been generated at any point in a player’s career and the extent to which affiliation-based external status specifically impacted career outcomes is not clear. To address this, career outcomes need to be analyzed after players receive affiliation-based externals status (i.e., wins the Stanley Cup). Although it is clear when status is acquired by individuals, players win the Stanley Cup at different points in their careers (i.e., some players win early in their careers, others win later). This makes it difficult to have a traditional pre post design with a control group as there is not a standardized point in which players either win or become finalists.

To address this challenge, we utilized a matching technique. In these analyses, award winners were matched at the time they won the award with a non-winner of commensurate ability and experience. Matching controls for some of or all of the confounding influence of pre-treatment control variables in observational data by creating a sample that did not receive the treatment (i.e., did not win the Stanley Cup) that is comparable on all observed levels to the sample that did receive the treatment (i.e., those that won the Stanley Cup). Then, outcomes after the players were matched can be compared to determine if affiliation-based external status generates any differences in career outcomes.

This work utilizes coarsened exact matching (CEM) methods to match Stanley Cup winners. CEM is a method for improving the estimation of causal effects by reducing imbalances in covariates between treated and control groups [27]. CEM is a monotonic imbalance-reducing matching method, which means that the variables the user wants to balance between the treated and the control groups is chosen by the user prior to completing any analyses [27]. The idea of CEM is to temporarily coarsen each variable into substantively meaningful groups, exact match on these coarsened data, and then retain only the original (un-coarsened) values of the matched data [27]. Users complete analyses on the un-coarsened data.

The first task was to choose a set of covariates to guarantee balance between the treatment group (i.e., players that won the Stanley Cup) and the control group (i.e., players that did not win the Stanley Cup). To match Stanley Cup winners with an appropriate comparison, players were matched on the number of years they have played in the NHL, their position, and their skill index. A player’s skill index is simply the number of games they’ve played plus their points. A skill index was calculated for each season players played, and a cumulative skill index was kept for each player. The winner’s cumulative skill index for the year they won the Stanley Cup was recorded and then they were matched with a player in the same season of their career, who played the same position, and had a similar skill index. For example, let’s say Player X is a forward who won the Stanley Cup in his second season. At the time of his second season, he had 50 points and 140 games played, equaling a cumulative skill index of 190. Player X would then be matched with a player that has not won the Stanley Cup, that is also a forward, in his second season that has a skill index of 190.

Exact matching was used where possible. Due to the number of player positions and the number of years in the league, exact matching could be used for those categories. Skill indexes were more variable and as such, we had to create predetermined cut-offs for matching. The predetermined cut-off for bins was 50 points on the skill index. Binning for variables should be customized according to the distribution of the variable and the bins for the variable should be created as to ensure the closest matches possible without losing a large proportion of the sample [28]. All winners, except for one who was excluded from the dataset for not having a comparable match, received a match. The mean skill index for winners was 492 and the mean skill index for non-winners was 491. Of the 73 pairs, 18 were perfect matches, 46 matches were within three points of each other, 56 matches were within five points, 64 matches were within ten points, and the remaining nine were within 43 points. From the matched players, two groups were created: one with players who have won the Stanley Cup and the other consisting of those who had not won the Stanley Cup. Four t-tests were conducted to ensure that there are not any significant differences between winners and non-winners on any of the indexes used in the matching process (Table 6). None of the tests were significant, lending support for our matching method.

**Table 6:** Results of t-tests and descriptive statistics analyzing matching method.

	Winners		Non-Winners						
Outcome	M	SD	M	SD	n	*Sig*	*df*	*d*	95% CI for Effect Size
Year in Career	5.82	3.56	5.82	3.56	146	= 1	144	0.00	-.32, .32
Games Played	351.99	255.26	349.73	261.45	146	= .96	144	0.01	-.32, .33
Points	140.23	156.61	140.95	144.16	146	= .98	144	0.00	-.33, .32
Skill Index	492.23	389.77	490.67	386.54	146	=. 98	144	0.00	-.32, .33

The effects of winning the Stanley Cup (between subject: winner vs. non-winner) and Time (within subject: pre-treatment vs. post-treatment) on Opportunities (measured by number of Games Played and Total Ice Time) were examined using two between versus within 2x2 ANOVAs. The effects of winning the Stanley Cup (between subject: winner vs. non-winner) and Time (within subject: pre-treatment vs. post-treatment) on productivity (measured by Points) was also examined using another between versus within 2x2 ANOVA. Descriptive statistics are reported in Tables 7–9.

**Table 7:** Means and standard deviations for GP as a function of a 2 (Won Cup) X 2 (Time) design.

	Time					
	Pre		Post		Marginal	
WonCup	*M*	*SD*	*M*	*SD*	*M*	*SD*
No	349.73	261.45	257.62	269.01	303.67	268.35
Yes	351.99	255.26	420.49	255.13	386.24	256.63
Marginal	350.86	257.48	339.05	273.74		

*Note*. *M* and *SD* represent mean and standard deviation, respectively. Marginal means for one factor are the means for that factor averaged across all levels of the other factor.

**Table 8:** Means and standard deviations for TOI as a function of a 2 (Won Cup) X 2 (Time) design.

	Time					
	Pre		Post		Marginal	
WonCup	*M*	*SD*	*M*	*SD*	*M*	*SD*
No	5861.25	5217.32	4526.68	5173.78	5193.97	5220.76
Yes	6167.19	5416.94	7183.53	5062.70	6675.36	5249.52
Marginal	6014.22	5301.92	5855.11	5272.16		

*Note*. *M* and *SD* represent mean and standard deviation, respectively. Marginal means for one factor are the means for that factor averaged across all levels of the other factor.

**Table 9:** Means and standard deviations for Pts as a function of a 2 (Won Cup) X 2 (Time) design.

	Time					
	Pre		Post		Marginal	
Cup Winner	*M*	*SD*	*M*	*SD*	*M*	*SD*
No	140.95	144.16	101.40	152.92	119.53	149.28
Yes	140.23	156.61	174.86	196.32	159.41	179.94
Marginal	140.59	149.99	138.13	179.19		

*Note*. *M* and *SD* represent mean and standard deviation, respectively. Marginal means for one factor are the means for that factor averaged across all levels of the other factor.

First, the main effect of winning the Stanley Cup on games played was examined. The main effect of winning the Stanley Cup was significant, *F* (2, 288) = 14.29, *p* = .000, partial η^2^ = .05, 90% CI [.02, .09]. The marginal mean for Stanley Cup winners was 386.24 and the marginal mean for non-winners was 303.67, revealing that Stanley Cup winners played significantly more games in their careers than non-winners. This result is consistent with previous analyses indicating that winners have more games played over their careers than non-winners.

**Fig 1:**
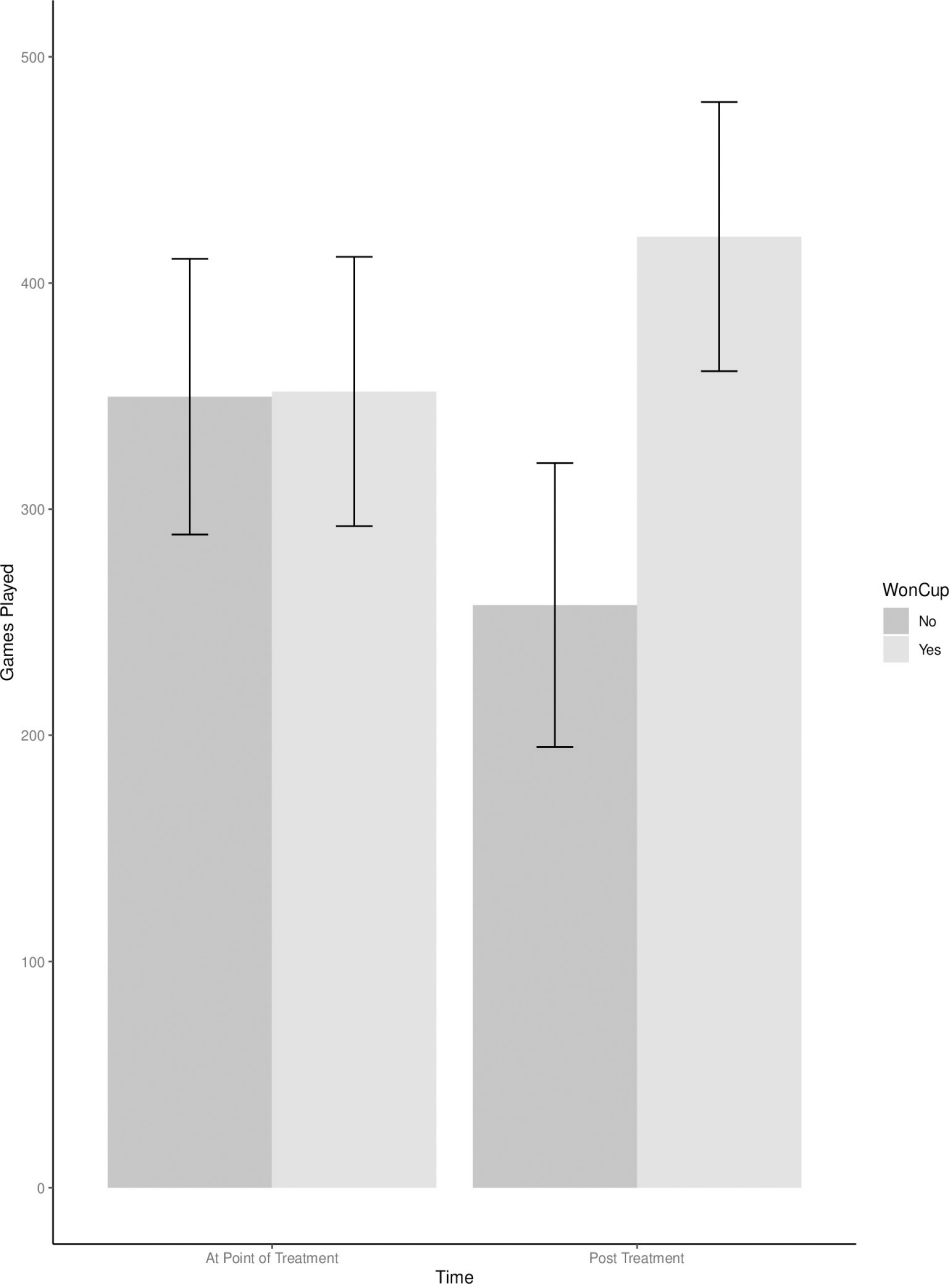
Group means for the interaction of games played as a function of a 2 (Won Cup) X 2 (Time) design.

The main effect of time (pre/post treatment) was also statistically significant, *F*(2, 288) = 4.57, *p* = .033, partial η^2^ = .02, 90% CI[.00, .05]. Examining the main effect of time (pre/post treatment) reveals the pre-treatment marginal mean was 350.86 and the marginal mean post-treatment was 339.05, suggesting players played more games pre-treatment than post treatment. The interaction between winning the Stanley Cup and time was also significant, *F*(1, 288) = 6.95, *p* = .009, partial η^2^ = .02, 90% CI[.00, .06], (Fig 1 and Table 10). The significant interaction in Fig 1 reveals that there was a change in the number of games played at time two (i.e., after winning the Stanley Cup), such that those who won the Stanley Cup (M = 420.49) played more games than those who had not won the Stanley Cup (M = 269.62) post-treatment. This result is consistent with the expectation that winning results in more opportunities for winner compared to non-winners.

**Table 10:** Fixed-Effects ANOVA results using games played as the criterion.

Predictor	Sum of Squares	*df*	Mean Square	*F*	*p*	_partial_ η^2^	_partial_ η^2^ 90% CI [LL, UL]
(Intercept)	4844734.74	1	4844734.74	71.52	.000		
WonCup	968302.05	1	968302.05	14.29	.000	.05	[.02, .09]
Time	309672.44	1	309672.44	4.57	.033	.02	[.00, .05]
WonCup x Time	470806.93	1	470806.93	6.95	.009	.02	[.00, .06]
Error	19510225.01	288	67743.84				

*Note*. LL and UL represent the lower-limit and upper-limit of the partial η^2^ confidence interval, respectively.

**Fig 2:**
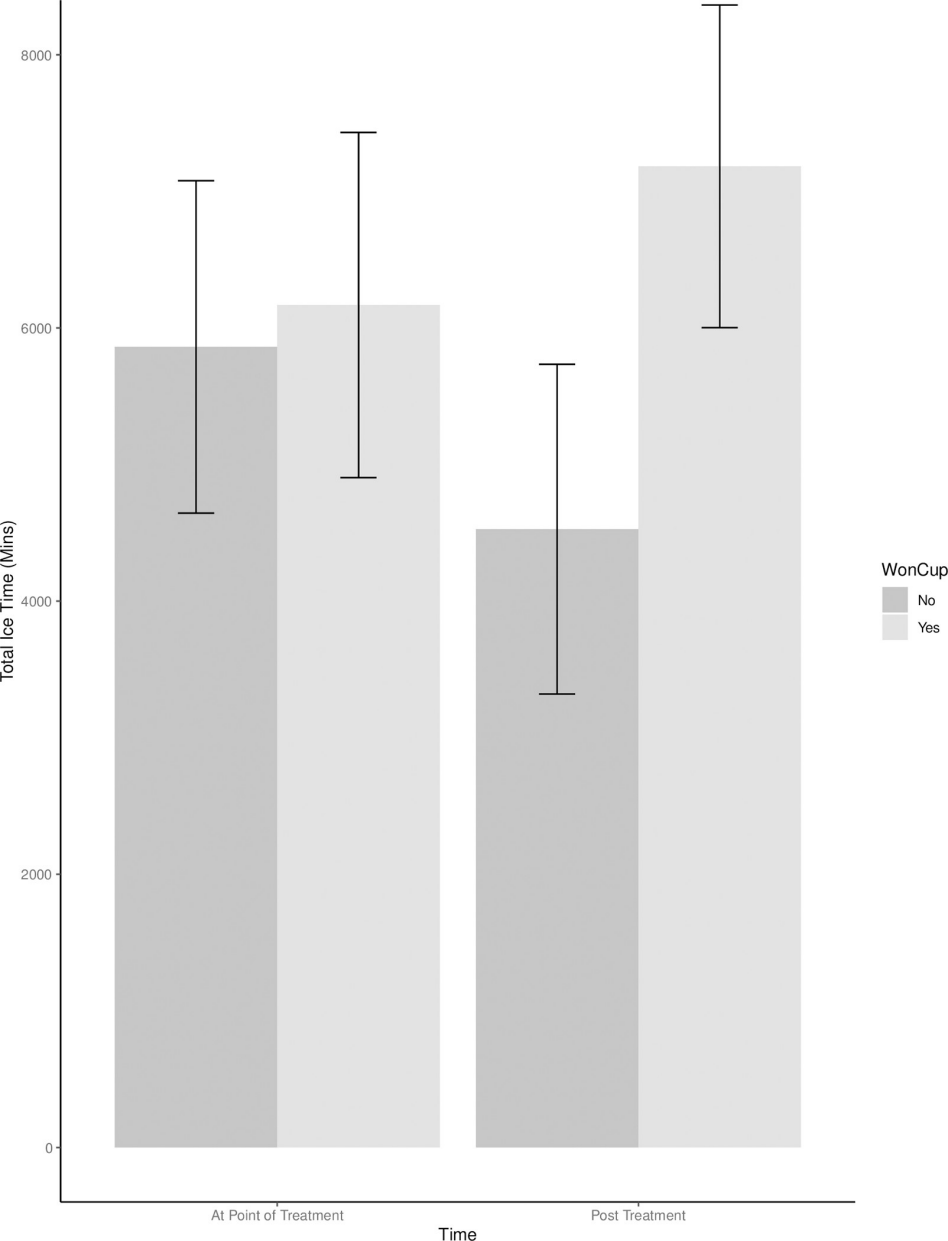
Group means for the interaction of total ice time as a function of a 2 (Won Cup) X 2 (Time) design.

The effects of winning the Stanley Cup on total ice time was then examined. The main effect of winning the Stanley Cup was significant, *F* (2, 290) = 9.46, *p* = .002, partial η^2^ = .02 90% CI [.01, .07]. The marginal means of winning the Stanley Cup (6675.36) was greater than the marginal mean for non-winners (5193.97), indicating winners play significantly more minutes in their career overall. The main effect of time (pre/post treatment) was not significant, *F*(2, 288) = 2.39, *p* = .123, partial η^2^ = .01, 90% CI[.00, .03]. The marginal means pre-treatment (6014.22) appear to be greater than the marginal mean post-treatment (5855.11), indicating that players did not play different amounts before and after the treatment. The interaction between winning the Stanley Cup and time was not significant, *F*(1, 288) = 3.70, *p* = .055, partial η^2^ = .01, 90% CI[.00, .04], (Fig 2 and Table 11). Although the pattern of the interaction is consistent with winners playing more minutes after the award (i.e., treatment) than non-winners, and *p* = .055, the differences post treatment were not statistically significant at *p* < .05.

**Table 11:** Fixed-effects ANOVA results using TOI as the criterion.

Predictor	Sum of Squares	*df*	Mean Square	*F*	*p*	_partial_ η^2^	_partial_ η^2^ 90% CI [LL, UL]
(Intercept)	1495833982.25	1	1495833982.25	54.91	.000		
WonCup	257647962.33	1	257647962.33	9.46	.002	.03	[.01, .07]
Time	65008499.51	1	65008499.51	2.39	.123	.01	[.00, .03]
WonCup x Time	100863189.92	1	100863189.92	3.70	.055	.01	[.00, .04]
Error	7845296784.79	288	27240613.84				

*Note*. LL and UL represent the lower-limit and upper-limit of the partial η^2^ confidence interval, respectively.

**Fig 3:**
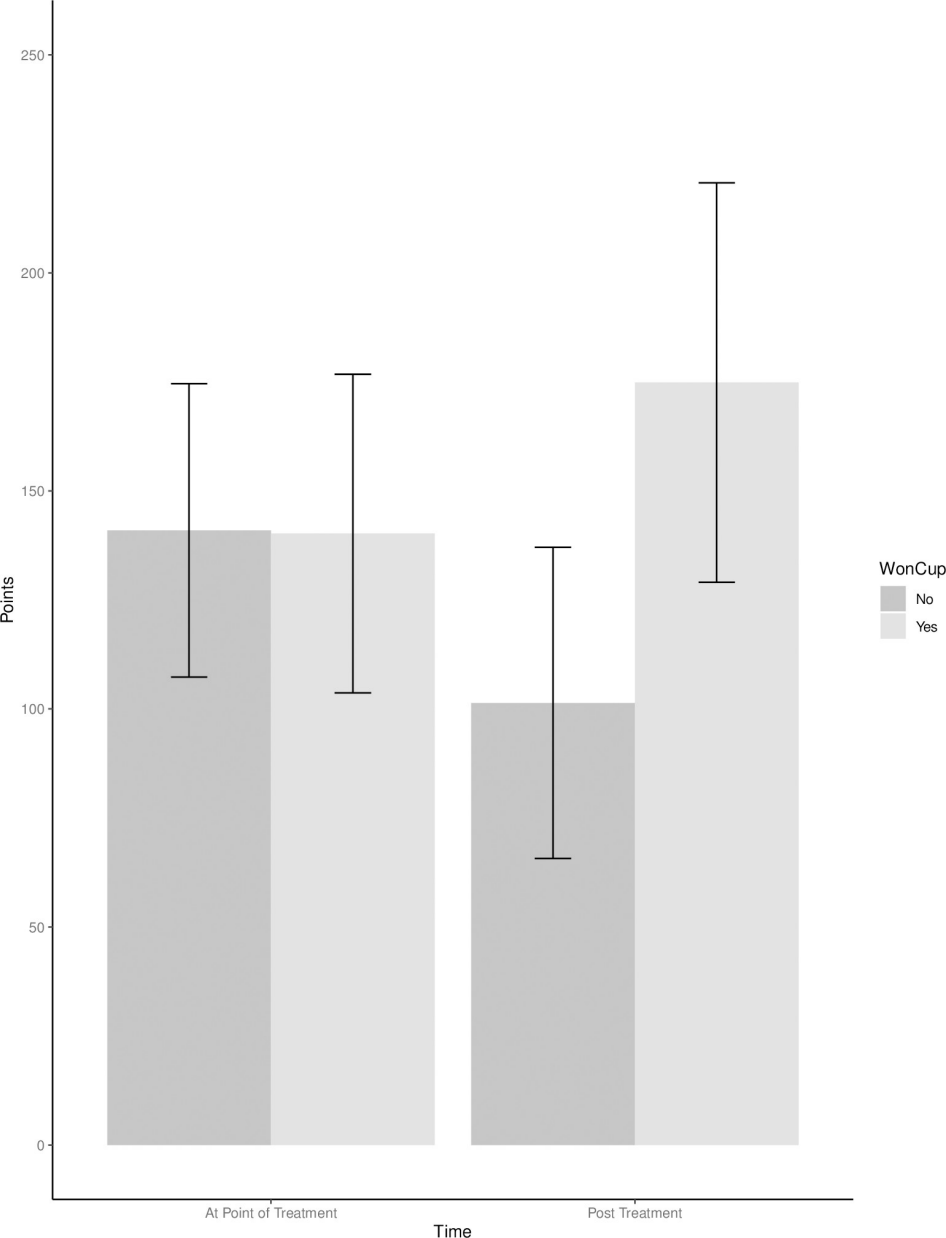
Group means for the interaction of points as a function of a 2 (Won Cup) X 2 (Time) design.

Next, the effects of winning the Stanley Cup on productivity, as measured by points, was examined. The main effect of winning the Stanley Cup was significant, *F* (2, 290) = 7.35, *p* = .007, partial η^2^ = .02 90% CI[.00, .06]. The marginal means of winning the Stanley Cup (157.55) was greater than the marginal mean for non-winners (121.17), indicating winners had significantly more points in their careers than non-winners. The main effect of time was not significant, *F*(2, 288) = 2.13, *p* = .146, partial η^2^ = .01, 90% CI[.00, .03]. The marginal mean pre-treatment (140.59) is slightly greater than the marginal mean post-treatment (138.13), but the difference is not statistically significant. The interaction between winning the Stanley Cup and points was not significant at *p* < .05, *F*(1, 288) = 2.61, *p* = .054, partial η^2^ = .01, 90% CI[.00, .04], (Fig 3 and Table 12), although the pattern of the interaction is consistent with winners acquiring more points after the treatment than non-winners.

**Table 12:** Fixed-effects ANOVA results using points as the criterion.

Predictor	Sum of Squares	*df*	Mean Square	*F*	*p*	_partial_ η^2^	_partial_ η^2^ 90% CI [LL, UL]
(Intercept)	750542.52	1	750542.52	28.00	.000		
WonCup	196998.42	1	196998.42	7.35	.007	.02	[.00, .06]
Time	57087.46	1	57087.46	2.13	.146	.01	[.00, .03]
WonCup x Time	100418.58	1	100418.58	3.75	.054	.01	[.00, .04]
Error	7720790.93	288	26808.30				

*Note*. LL and UL represent the lower-limit and upper-limit of the partial η^2^ confidence interval, respectively.

Together, these results suggest that when matching winners with an equivalent player with respect to tenure and ability at the time of the win, winning the Stanley Cup results in an increased number of games played post win. Although the interactions for time on ice and points were in the expected pattern, the interactions were not statistically significant at *p* < .05 (*p* = .055 and *p* = .054, respectively).

### Is there an indirect effect on productivity through opportunities?

**Fig 4:**
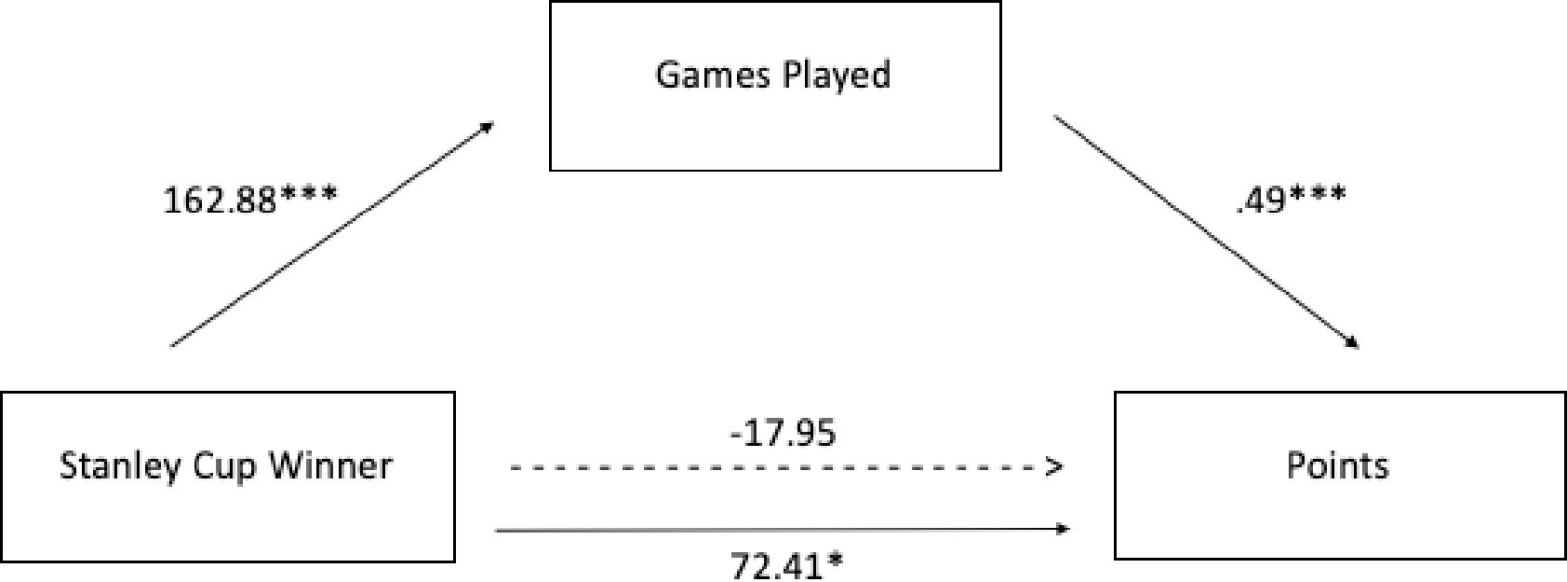
Unstandardized coefficients for the relationship between being a Stanley Cup winner and points as mediated by games played.

Although the above results demonstrated that there was not a statistically significant direct effect of status on productivity following the onset of status, the Matthew Effect posits that awards can generate status that results in more opportunities, which then lead to resource gains [4]. Thus, it is possible that opportunities may act as a mechanism for a possible indirect relation between status and productivity. In the next analysis, we examine if status has an indirect effect on productivity through opportunity. To do so, a mediation analysis was conducted to examine the possible impact of opportunity in facilitating resource gain. Using the matched paired dataset, the mediation analysis examined whether being a Stanley Cup winner (*X;* compared to matched non-winners at time of the win) affects the number of points (*Y)* through the number of games a player has played (*M*). In this scenario, *c* is the zero-order relation between being a Stanley Cup winner and points (*Y)*. The *indirect effect* is the effect of being a Stanley Cup winner on points that is mediated through games played (the product of the a and b paths, a*b). To test the statistical significance of the indirect effect, the *mediation* package was utilized in R. Results of these analyses are depicted in Fig 4. As Fig 4 illustrates, the regression coefficient between being a Stanley Cup winner and points and the regression coefficient between games played and points were significant. The model shows a significant indirect effect of games played on the relationship between winning the Stanley Cup and points (90.36 [39.13, 143.46], *p* < .001) (Table 13). The Average Causal Mediation Effect (ACME), or indirect effect, represents the effect of the mediator (i.e., games played) alone on productivity and is significant. The average direct effect (ADE) describes the direct effect of being a Stanley Cup winner on points and is insignificant. The total effect (ACME + ADE) describes the total effect of the treatment on the outcome and is also significant. Prop. mediated describes the proportion of the effect of being a Stanley Cup winner on points that goes through games played. It is calculated by dividing the ACME by the total effect and is also significant. Thus, the indirect, or mediation effect, of games played was statistically significant.

**Table 13:** Causal mediation analysis for the relationship between being a stanley cup winner and points as mediated by games played.

Nonparametric Bootstrap Confidence Intervals with the Percentile Method				
	Estimate	95% CI Lower	95% CI Upper	p-value
ACME (control)	79.277	35.967	126.50	<2e-16***
ACME (treated)	101.449	40.496	167.79	<2e-16***
ADE (control)	-29.037	-58.332	-1.50	0.040*
ADE (treated)	-6.864	-58.332	46.07	0.792
Total Effect	74.865	13.178	130.88	0.012 *
Prop. Mediated (control)	1.095	0.542	3.21	0.012*
Prop. Mediated (treated)	1.401	0.997	3.27	0.012*
ACME (average)	90.363	39.133	143.46	<2e-16***
ADE (average)	-17.951	-51.904	20.76	0.356
Prop. Mediated (average)	1.248	0.784	3.14	0.012 *

Signif. codes: 0 ‘***’ 0.001

‘**’ 0.01

‘*’ 0.05 ‘.’ 0.1 ‘ ‘ 1.

Sample Size Used: 146.

Simulations: 1000.

*Upsilon(*) was then utilized to determine the effect size of the indirect effect. The effect size is a method for quantifying explained variance in mediation analysis, and is interpreted as the variance in Y accounted for jointly by M and X that corrects for spurious correlation induced by the ordering of variables [29]. In the current analyses, is the estimated variance in points explained by winning the Stanley Cup through games played and was 0.063 with a 95% CI [0.01, 0.16]). This indicates that 6% of the variance in points is explained through the indirect effect of winning the Stanley Cup through games played.

## Study 1: Discussion

Study 1 sought to answer (1) if individuals with affiliation-based external status have more opportunities across their careers than individuals that do not have the same affiliation-based external status, (2a) if those with affiliation-based external status are more productive across their careers than individuals that do not have the same affiliation-based external status, and (2b) if opportunity partially explain differences in career productivity between those with affiliation-based external status and those without it. Overall, the results provide some insight on the effect of status on career outcomes and generally support the Matthew Effect. The most consistent finding is that winners get more opportunities than non-winners. This result was found on aggregate, across careers, and was also found to accrue post win. Even as the comparison group became increasingly similar to winners (i.e., finalists, matched pairs), winners were found to accrue more opportunities than non-winners. Also, when there was evidence that winning did not directly increase productivity, productivity was found to increase indirectly through the acquisition of more opportunities. These findings support the idea that affiliation-based external status, generated by an award win, bequeaths winners with advantages above and beyond winners’ positive endogenous characteristics. Additionally, the above findings indicate that opportunity is a key outcome of status gain.

### Supplementary analyses 1

A main finding from the focal study reveals that winners get more opportunities than non-winners across their careers. Supplementary analyses were conducted to investigate possible mechanisms underlying opportunity differences and to extend our findings by testing another ancillary tenet of the Matthew Effect, which suggests that additional opportunities lead to resource gains. To investigate how status from winning an award may operate to create more resources for winners, we examined the timing of when winners acquire more resources and a possible mechanism of this acquisition. Given the expected mechanism of cumulative advantage operating over time, we wanted to examine when these opportunity differences may begin to arise and if they may be the result of formal work arrangements. Specifically, we wanted to determine (a) if winners procure enhanced opportunities (i.e., length of contract, total dollar amount of contract) immediately following their win and (b) if these advantages are formally incorporated into contracts. To examine these questions, we examined if winners were able to secure better contracts than finalists immediately following their win and loss, respectively. If winners receive better contracts immediately following their win, this would provide evidence for *how* cumulative advantage may operate over ones career. Namely, immediate advantages in resources codified in improved contractual agreements, which unfold over one’s career, and result in large differences at the conclusion of one’s career.

### Participants and procedure

Consistent with our focal study, NHL players were used as participants and the Stanley Cup was considered a marker of affiliation-based external status. However, a new dataset had to be created. NHL contracts can be different lengths, varying from a single year to as many as 12 [30]. The Stanley Cup typically concludes in late May or early June. There is no guarantee that a player’s contract will be completed immediately after winning the Stanley Cup. For example, a player could sign a 7-year contract in 2020 and win the Stanley Cup in 2021. As such, the player was already committed to 6 more years with their team and winning the Stanley Cup would therefore not have any bearing on their contract. To examine if winners immediately benefited from winning the Stanley Cup, analyses must be completed on players whose contracts expired immediately after their Stanley Cup appearance. Very few players from the matched dataset fit this requirement, so we could not use the dataset utilized in the focal study.

As such, a new dataset was created for our first supplementary analysis. We chose to compare winners to finalists as finalists are a group that, beyond matched data, most closely resemble winners. We utilized an online hockey salary database [31] for player transaction information. We recorded all winning teams’ and finalist teams’ signings *the year following their Stanley Cup win (i*.*e*., *winners) or Stanley Cup appearance (i*.*e*., *finalists)*. Specifically, we recorded individual player’s previous contracts and the newly negotiated contract’s length in years, the contract’s Average Annual Value (AAV; total compensation earned per year), and Total Salary (total career compensation). Of note, NHL salary data is not readily available prior to 2007. As such, data from Stanley Cup Finals from 2007–2019 was recorded. This resulted in contract data from 176 players (88 finalists, 88 winners).

### Measures

#### Affiliation-Based Status

Indicated whether a player had previously won the Stanley Cup (= 1) or whether they had not won the Stanley Cup (= 0).

#### Contract Length

The length of a player’s contract measured in years.

#### Total Salary

The total compensation for a player at the NHL level that includes signing bonuses, but not performance bonuses, measured in 2020 USD.

Average Annual Value (AAV). **A player’s total salary plus signing bonuses (measured in 2020 USD) of a contract divided by the contract’s length.**

#### Analytic approach

To test if winners received better contracts immediately following their win compared to finalists, linear regressions were conducted using player’s salary data from their contract prior to their Stanley Cup win or appearance to predict their contract immediately following their Stanley Cup win or appearance. This allowed us to both control for previous contract characteristics and generate residuals. Residuals are a measure of how much each player’s observed contract differs from what was predicted based on their contract from before their Stanley Cup win or appearance. Residuals from winners and finalists for their length, AAV, and total salary were saved and compared. If winners are found to have significantly greater residuals (e.g., earned more money than expected based on their previous contracts) than finalists, it would suggest that affiliation-based external status results in immediate, tangible resources. All comparisons were conducted with a series of paired *t*-tests.

## Results

A test of residuals reveal that Stanley Cup winners (*M* = -.26) did not receive significantly longer contracts than expected after winning the Stanley Cup than finalists (*M* = .26), *t*(174) = 1.5, *p* = .1356, did not (*M* = -2004581) receive significantly more total salary than expected after winning the Stanley Cup than finalists (*M* = 2004581), *t*(174) = 1.67, *p* = .1, and did not (*M* = -261842.9) receive significantly greater AAV than expected after winning the Stanley Cup than finalists (*M* = 261842.9), *t*(174) = 1.86, *p* = .1356. Altogether, these results suggest that an award win, and corresponding affiliation-based external status, did not result in the acquisition of immediate, formally conveyed benefits.

### Supplementary analyses 2

Supplementary Analyses 1 did not find any opportunity differences in the form of improved contracts immediately after affiliation-based external status acquisition. To follow up on the findings of the focal study, we extended our investigation to examine how affiliation-based external status may operate near the end of individuals’ careers. Professional sports leagues are highly competitive and there is a high rate of turnover from these leagues. As a consequence, a sign of player success is their career longevity. According to one analysis, in the NHL, bottom players play an average of 2 years, average players play 4.5 years, and the top players play an average of 12 years [32]. As a result, the ability to continue to play in the NHL represents a vital and foundational source of opportunity. In Supplementary Analyses 2, we sought to investigate if affiliation-based external status affords those with status more access to career extension opportunities.

In team settings, team output is observable and tangible, but it can be difficult to identify the distinct contributions of individual team members [33]. In team situations, the Matthew Effect suggests that high status team members tend get more of the credit for team performance, compared to less eminent team members [4]. More recent work has found that, in addition to getting more credit for good outcomes, more eminent members are punished less for bad outcomes when compared to less esteemed team members [33]. In light of the enhanced visibility of high-status team members, we investigated if status results in a player’s value at the end of their careers being differentially appraised. We inquire if status acts as a protective factor, such that the performance of those with status may be evaluated more favourably than those without status. As such, players with status may continue to get opportunities at the end of their careers in situations where players without status may not, as a result of more favourable evaluations.

Given the competitive nature of the NHL and limited roster space, teams are incentivized to remove unproductive players. Consequently, a marker of a player’s value is how much opportunity (i.e., ice time) a player receives by their teams within games (i.e., ATOI). Organizations allocate opportunities in a way that they feel gives them the best chance to be successful. As such, more valuable players typically get more ice time, or more time to influence the outcome of the game, than players that are not as valuable. Thus, as players’ playing time decreases in comparison to their average career ice time, we expect that there will be an increase in the likelihood of them exiting the league as they are being seen as less valuable by their organizations. However, if status acts as a protective factor and results in more favourable subjective evaluations of performance, we expect that players with status will not be subject to the same fate as those without status and decreased playing time will not result in an increased likelihood of exiting the league. Specifically, we examined if being a Stanley Cup winner alters the probability of exiting the league and ending ones’ career as a function of how much in game playing time they had the previous season.

## Participants and procedure

To investigate this question, we revisited the coarsened exact matching (CEM) dataset that was used to match Stanley Cup winners in the focal study (see focal for complete details on participants and procedures). In total, this analysis utilizes 73 matched pairs of Stanley Cup winners and non-winners. Each Stanley Cup winner was matched with a non-winner with a similar skill index, that played the same position, and had played in the league for the same amount of years at the point when the winner won.

### Measures

#### Affiliation-Based Status

Indicated whether a player had previously won the Stanley Cup (= 1) or whether they had not won the Stanley Cup (= 0).

### Average Time on Ice (ATOI)

The average amount of time the player spent on the ice in the games he played (calculated by dividing each player’s seasonal time on ice by their total games played).

#### Exiting the League

Indicated whether a player was in his last season (= 1) or not (= 0).

#### Analytic approach

To answer the above question, we used a multilevel logistic regression using the nlme package in R [34]. The model sought to test if it was possible to predict the year in which player’s exited the league with players’ ATOI. Data was collected across individual players’ careers, individual seasons are nested within players, necessitating multilevel regression. Average total ice time was centered around their mean before computing the interaction term, and all terms were entered into the model together.

## Results

**Fig 5:**
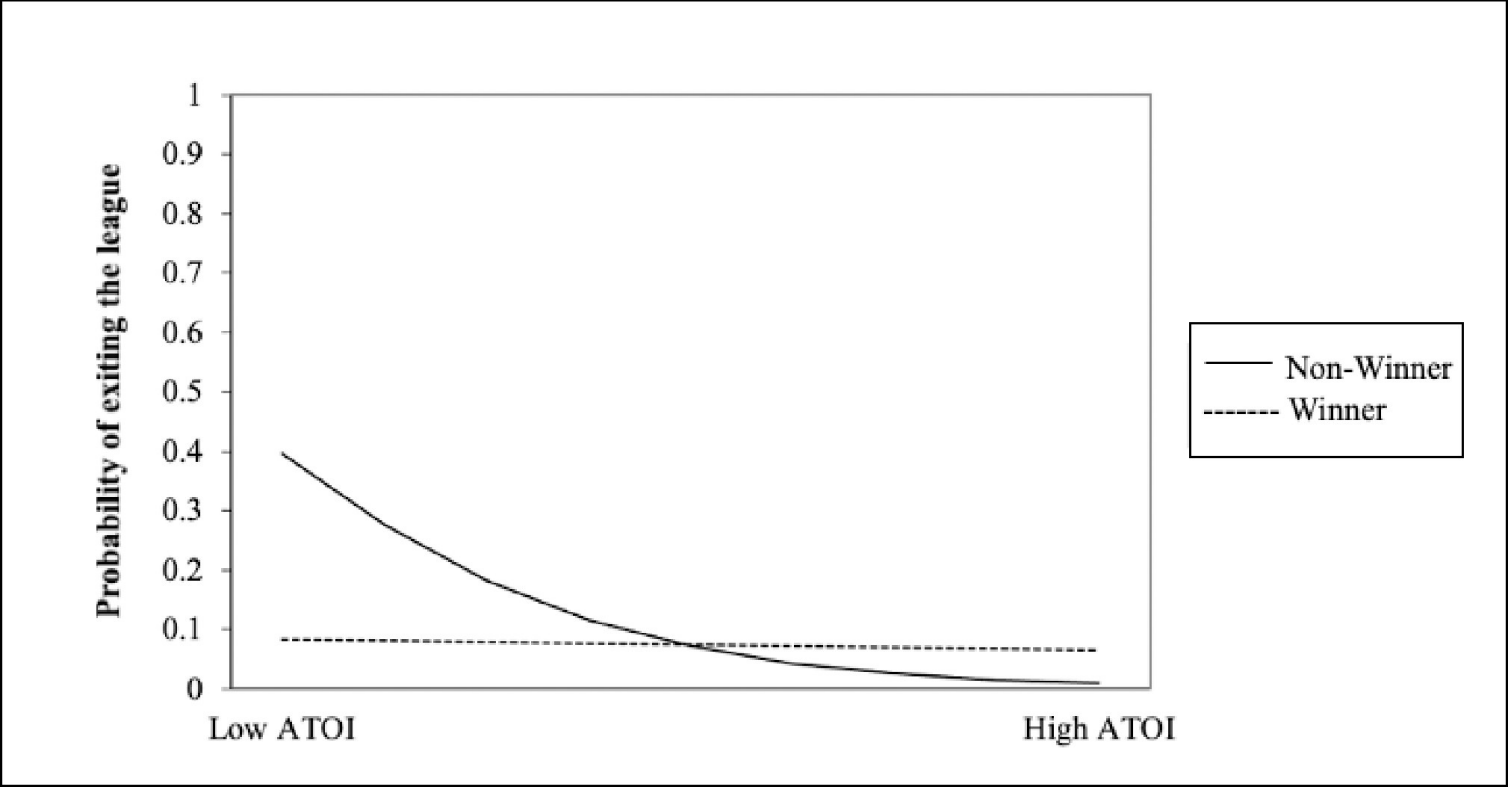
Multilevel logistic regression results predicting exiting or not exiting the league with average total ice time (ATOI).

The results indicated a significant interaction between ATOI and affiliation-based external status in predicting whether a player will end his career, *b* = .006, SE = .005, *p* < .00 (Fig 5). The significant interaction suggests that the effect of ATOI is different on the probability of exiting the league, depending on if you are a Stanley Cup winner. For Stanley Cup winners, the odds of exiting the league do not significantly change if you have high or low ATOI. For non-winners, the odds of exiting the league are much higher if a player has low ATOI compared to high ATOI. Thus, players with status appear to stay in the league longer, irrespective of how many minutes they are playing, whereas individuals without status need to have a high ATOI to stay in the league. This finding provides some evidence that the observed differences in increased opportunities for those with affiliation-based external status occurs from more favourable evaluations at the end of one’s career.

## General discussion

The current work investigated if the status achieved from winning an award can be directly translated into positive career outcomes. Our primary goals were to identify if there was an effect of winning an award on career outcomes, irrespective of the positive endogenous characteristics of award winners, and to investigate the Matthew Effect, particularly the role opportunity may play in generating positive outcomes. To do so, we identified an award that confers affiliation-based external status and is awarded to individuals who possess a range of positive endogenous characteristics prior to receiving the award: the Stanley Cup. In our investigation, we found that winning an award that bestows affiliation-based external status can uniquely generate positive differences in career outcomes for recipients. Specifically, we found that winning an award was associated with increased opportunities after the win by comparing winners to non-winners, finalists and individuals who were of equal standing prior to receiving the award (i.e., matched pairs). We found, in comparing winners and matched non-winners, that winning an award is not directly associated with increased productivity. However, productivity was found to increase indirectly through the acquisition of more opportunities. In supplementary analyses, we also found that winners appeared to benefit from different career extension opportunities compared to non-winners. Together, this work provides support for the Matthew Effect, whereby affiliation-based external status, bestowed by an award win, is associated with increased opportunities, which is associated with more productivity across careers.

By using professional sports data and a team award as a marker of affiliation-based external status, this work makes two important theoretical contributions. Initially, this work utilizes a novel approach to identify the effect of the change in status, without the conflation of individual differences. A team award was utilized and resulted in individuals with variable skill and productivity levels that all enjoy affiliation-based external status. This allowed for naturally occurring control groups, where one can find players from winning and non-winning teams of equal ability. Thus, players with affiliation-based external status were matched with players of commensurate ability without affiliation-based external status and comparisons could be made post-award win to determine the impact of affiliation-based external status. Further, tests of career opportunity and productivity measures were conducted without top performers in the dataset and significant differences still occurred. Taken together, these results suggest affiliation-based external status has the ability to exclusively generate differences for winners above and beyond winners’ positive endogenous characteristics.

Second, professional sports data provided a way to formally operationalize and investigate opportunity as a mechanism of the effect of awards on career productivity. Despite the theorized importance of opportunities in cumulative advantage, based on prior research, the role opportunities play in the Matthew Effect was not clear. Professional sports data offers statistics that very cleanly capture the construct of opportunities. A significant mediation with a small to medium effect size reveals that opportunity acts as a mechanism of the Matthew Effect and is a significant contributor to the relationship between winning an award and productivity.

This finding spurred further investigation into the role of opportunity and the roles it plays into the cumulative advantage process. An award win that conveys affiliation-based external status does not result in the acquisition of immediate and formal benefits by way of improved contracts following their win. However, further work reveals that the amount of opportunities a player receives each game and the probability a player exits the league depends on whether the player is a Stanley Cup winner. Thus, status appears to act as a protective factor, where winners continue to be afforded more opportunities when their on-ice value declines while their peers without status do not receive the same opportunities. This result suggests that players with status are either evaluated more positively or organizations perceive other benefits, aside from on ice performance, when deciding to retaining those with status.

Practically, these results suggest that status achieved from winning an award can be translated into tangible benefits, consistent with the Matthew Effect. Most consistently, those with status receive more opportunities than their peers of equal ability. Surprisingly, those with higher status are not necessarily more productive than their peers and small differences in productivity can often be attributed to receiving increased opportunities. Together, these results suggest that status may be an unreliable predictor of future productivity. Consequently, those in positions where they are making selection decisions for funding, jobs, or promotions may benefit from the use of performance related predictors instead [e.g., general mental ability, personality; 35,36]. Further, funding bodies, awards committees, HR personnel etc. should recognize the potential for impact of their awards (i.e., increased opportunities for winners) and strive to make decisions in the most just way possible. Steps could include making professional societies and funding bodies aware of the potential for biases to influence selection and reward decisions and reviewing award/promotion criteria to ensure sure they are strongly related to future performance.

## Limitations and future directions

There are several limitations of the dataset we used. This research needed to be done with archival data, as it is not possible to manipulate awards and measure career outcomes in a laboratory. It was also not possible to randomly assign participants to conditions, manipulate these variables and study them on a large-scale career timeline.

The NHL could also be considered an exceptional case in the cumulative advantage literature. The average NHL salary was $2 690 000 USD or $3 3603 954 CAD in 2019–2020 [37] whereas the median income for American households was $70 784 USD in 2021 [38] Additionally, over 5 million people tuned into each Stanley Cup game in 2019 [39]. It could be possible that sports, specifically the NHL and Stanley Cup, are so different from other workplaces and workplace awards that the patterns of results might not remain consistent. However, the current research does suggest that cumulative advantage can occur in even elite contexts, where everyone has already attained very high status and are considered to be exceptional players. Thus, the effect could be more pronounced in non-elite contexts. Additional work needs to be completed in order to gain a clearer sense of the Matthew Effect in more typical workplaces.

Another limitation is our operationalization of status. Our designation of status is effectively a dichotomous operationalization of status versus no status. Due to sample size limitations, we were not able to capture additional distinctions with our operationalization; namely, some players winning multiple Stanley Cups throughout their careers, and some players being finalists as well as winners. In future research, it may be possible to have ordered categorical variables for different levels of status or perhaps operationalization that are more continuous in nature.

## Conclusion

This work explored the role of affiliation-based external status on career outcomes. By using a team award, we were able to extricate the effects of affiliation-based external status and mitigate effects of individual differences (e.g., talent, ability) on career outcomes. By using professional sports data, we were able to precisely capture and investigate opportunity and its role in the cumulative advantage process. Generally, this work provides support for the Matthew Effect, where affiliation-based external status, achieved by an award win, provides access to increased opportunities, which ultimately results in more productivity. Further, this work supports the practice of investigating specific mechanisms of the Matthew Effect as a means to broaden our understanding of the role of affiliation-based external status in the world of work.
